# The Emerging Role of Tertiary Lymphoid Structures as Predictive and Prognostic Biomarkers of Immunotherapy in Cancer

**DOI:** 10.1002/cam4.71728

**Published:** 2026-03-17

**Authors:** Lidia B. Medhin, Rodas Amanuel, Lydia Warburton, Leslie Calapre, Benhur Amanuel, Elin S. Gray

**Affiliations:** ^1^ Centre for Precision Health Edith Cowan University Joondalup Western Australia Australia; ^2^ School of Medical and Health Sciences Edith Cowan University Joondalup Western Australia Australia; ^3^ School of Medicine University of Western Australia Crawley Western Australia Australia; ^4^ Faculty of Health Science and Medicine Bond University Robina Queensland Australia; ^5^ Department of Medical Oncology Fiona Stanley Hospital Murdoch Western Australia Australia

**Keywords:** biomarker, immunotherapy, tertiary lymphoid structures

## Abstract

**Background:**

Immune checkpoint inhibitors (ICIs) have improved outcomes across several cancers, yet many patients do not respond, highlighting the need for robust predictive biomarkers. Tertiary lymphoid structures (TLS), ectopic lymphoid aggregates that support local antigen presentation and adaptive immune activation, have emerged as potential indicators of favourable prognosis and immunotherapy responsiveness.

**Methods:**

This review summarises current clinical and translational evidence examining the prognostic and predictive value of TLS in solid malignancies. Studies assessing TLS presence, organisation, and biological function were identified through searches of major scientific databases and evaluated with respect to their association with patient outcomes and responses to ICIs.

**Results:**

Across multiple tumour types, TLS correlate with improved survival and enhanced anti‑tumour immune activity. TLS‑rich tumours typically show increased infiltration of effector immune cells and more inflamed tumour microenvironments. Several studies also indicate that TLS maturity, particularly the presence of germinal‑centre‑like features, strengthens their predictive value for ICI benefit. However, substantial variation exists in TLS assessment methods and definitions, limiting comparability and hindering translation into routine clinical use.

**Conclusions:**

TLS represent a promising biomarker for prognosis and immunotherapy response. Standardised evaluation methods and prospective clinical validation are essential to enable their integration into personalised treatment strategies.

## 1 Introduction

Tertiary Lymphoid Structures (TLS) are ectopic, immune aggregates that form in non‐lymphoid tissues in response to chronic inflammation, including within the tumour microenvironment (TME) [1]. They can be found either within tumours or in the peritumoural region [2]. The term ‘tertiary’ was first used in 1992 by Picker and Butcher to describe lymphoid structures in peripheral tissues [3], and later proposed by Takemura et al. in 2001, in their work on germinal centres in rheumatoid synovitis [4] (Figure 2).

Increasing evidence indicates that TLS influence antitumour immunity, particularly in the context of cancer immunotherapy [5]. As a result, TLS have gained attention as a potential prognostic and predictive biomarker. However, their clinical significance remains inconsistent across cancer types, highlighting substantial biological heterogeneity and limiting their immediate translational application [5].

Across tumours, TLS are associated with divergent clinical outcomes [6]. While their presence often correlates with improved survival and response to immunotherapy [7], they have also been linked with aggressive disease and poor prognosis [6]. These contrasting observations indicate that TLS are not uniformly immunostimulatory or immunosuppressive. Instead, their functional impact is determined by tumour‐specific immune environments, including TLS maturity, cellular composition, and spatial organisation within the TME.

Given the role of TLS in orchestrating local immune activity, several emerging strategies aiming to induce or enhance TLS formation within tumours have been pursued to boost anti‐tumour immunity [8, 9]. Understanding how TLS orchestrates tumour immunity could lead to the development of novel or improved therapies designed to activate the anti‐tumour immune response more effectively.

This review, therefore, explores the evolving field of TLS research, with a focus on its biomarker potential and its role in cancer treatment strategies.

## 2 Structural Organisation of TLS

TLS share key architectural and functional features with secondary lymphoid organs (SLOs), including compartmentalised B and T‐cell zones, antigen‐presenting cells, and specialised stromal elements, but they develop ectopically in response to sustained inflammatory signalling [10]. Unlike SLOs, TLS lack encapsulation and display considerable heterogeneity in organisation and immune function.

**FIGURE 1:**
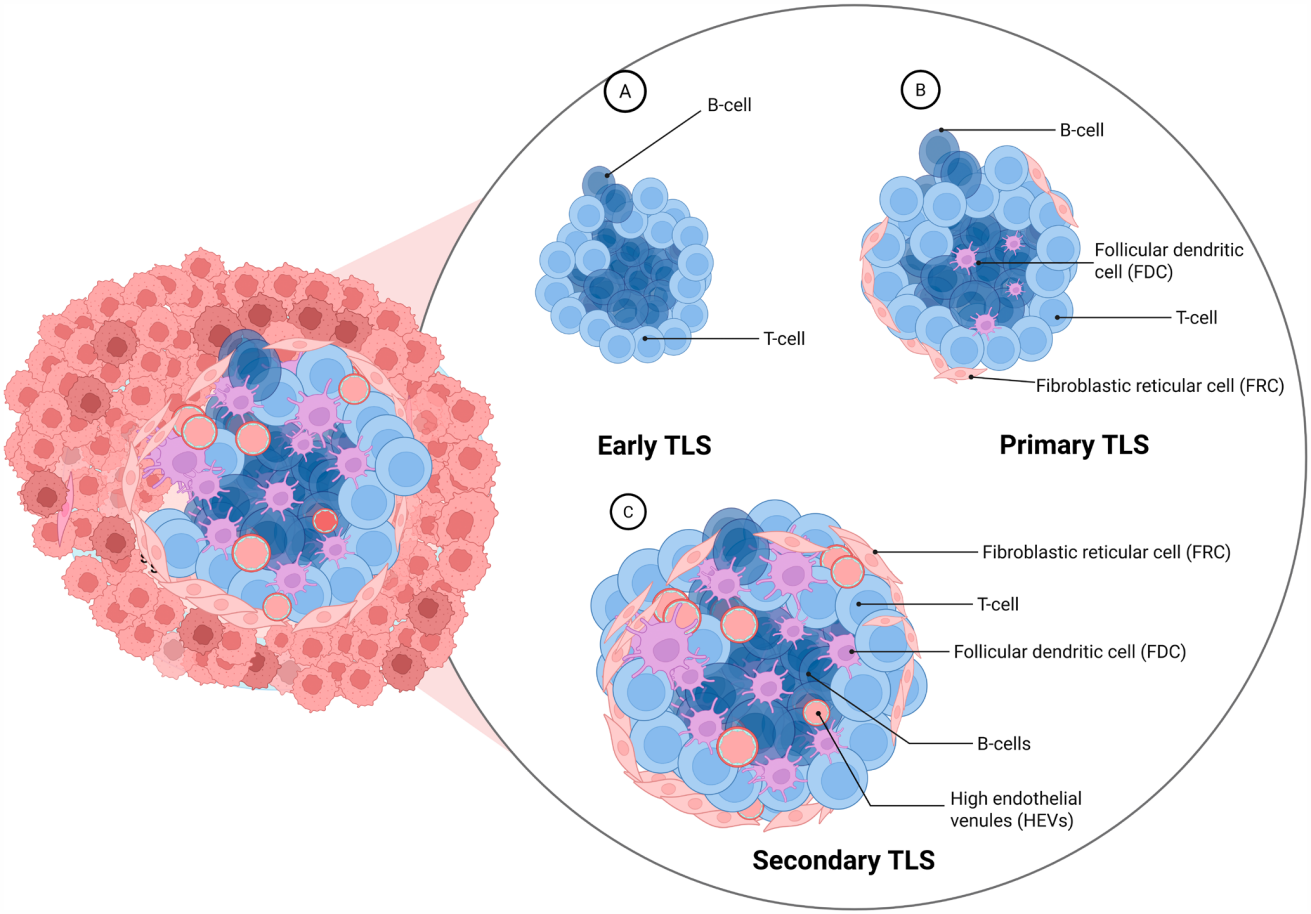
Developmental stages of TLS. Early TLS are marked by CD20^+^ B‐cell clusters and CD3^+^ CD4^+^ T‐cells, while primary TLS show more organisation with CD21^+^ immature follicular dendritic cells and fibroblastic reticular cells. Mature TLSs are characterised by CD23^+^ mature FDCs and HEVs (Image created with BioRender).

TLS are found at distinct developmental stages, which have been classified as early TLS, primary TLS, and mature TLS, each characterised by unique structural features and cellular compositions (Figure 1) [11]. Early TLS constitute initial immune cell aggregates, typically marked by dense CD20^+^ B‐cell clusters containing CXCL13^+^ cells, with consideration given to the presence or absence of interspersed CD4^+^ T‐cells [11, 12]. As the TLS mature, they exhibit enhanced organisation, including expression of CD21, a marker for immature follicular dendritic cells (FDCs) [13]. Further maturation leads to the development of secondary follicular TLS, which are often identified as structures with an additional network of CD23^+^ mature follicular dendritic cells [14].

Other studies have also defined maturity based on the presence of high endothelial venules (HEVs) expressing peripheral node addressin (PNAd), which signifies enhanced lymphocyte transmigration [15] or the presence of L1 cell adhesion molecule (L1CAM) expression in FDC [16]. Fibroblastic reticular cells (FRCs), which are usually identified by expression of podoplanin (PDPN) and BCL6‐expressing GC B‐cells, T‐follicular helper (TFH) cells also contribute to TLS architecture and function [17, 18]. Despite the debate on their classification, it is widely accepted that mature TLS support immune cell activation and recruitment, potentially influencing tumour immunity and therapeutic responses.

Chemokine and adhesion molecule networks are central to TLS development. Early characterization of TLS revealed key chemokines involved in T‐cell recruitment, such as CCL19, CCL21, CXCL13, CCL17, CCL22, and IL16, along with adhesion molecules including ICAM‐2, ICAM‐3, VCAM‐1, and MAdCAM‐1 as well as integrins, alphaL, alpha4, and alphaD [19].

Through these combined chemokine and molecular features, TLS actively modulate intratumoral immune cell infiltration rather than serving as passive immune aggregates. By establishing chemokine gradients, specialised HEVs and organised stromal networks, TLS promote the recruitment, retention, and spatial organisation of T cells, B cells, and antigen‐presenting cells within tumours [20]. Variations in TLS maturity and organisation, therefore, directly influence the density and functional state of infiltrating immune cells, providing for their heterogeneous immunological and clinical effects across cancer types [21].

TLS composition and organisation vary across cancer types, influencing immune functions and clinical outcomes. In melanoma and NSCLC, mature TLS are enriched in CD8^+^ cytotoxic T cells, CD4^+^ CXCL13^+^ T follicular cells, B cells, and mature dendritic cells, promoting strong antitumour immunity [22]. In contrast, TLS in luminal breast cancer and hepatocellular carcinoma are more frequently immature, composed of poorly organised T‐ and B‐cell aggregates with limited dendritic cell maturation and underdeveloped follicular dendritic cell networks associated with T‐cell exhaustion and immunosuppressive tumour microenvironments [23, 24]. These cancer type‐specific differences explain TLS heterogeneity in terms of differential immune activation and regulatory dynamics.

B lymphocytes play a central, yet context‐dependent, role within TLS. In mature TLS, memory B‐cells produce tumour‐specific antibodies and promote effective T‐cell activation, enhancing anti‐tumour immunity [25]. However, in an immature TLS, B‐cells might generate inhibitory factors and this can be supported by evidence linking immature TLS to reduced T‐cell activity in tumours [7, 23]. In addition, regulatory B‐cells (Bregs) and regulatory T‐cells (Tregs) are suggested to promote tumours through various phenotypes and immunosuppressive roles [26, 27, 28, 29].

Collectively, understanding TLS maturity stages and the dynamic interplay between TLS and the immune system in the tumour microenvironment is critical for advancing biomarker‐guided treatments and novel therapeutic strategies to improve patient outcomes.

## 3 TLS As a Prognostic Biomarker

**FIGURE 2:**
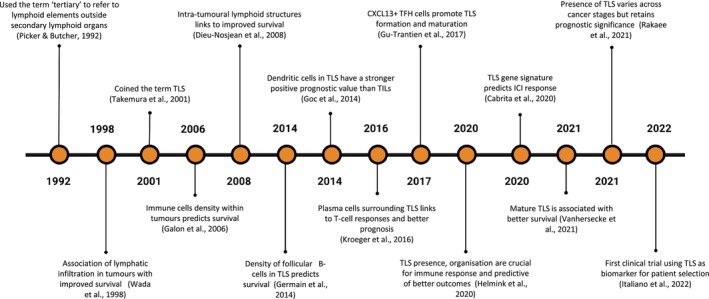
Timeline of key milestones in establishing TLS as prognostic and predictive biomarkers in cancer. ICI, immune checkpoint inhibitors; TILs, tumour‐infiltrating lymphocytes; TLS, tertiary lymphoid structures; TFH, T follicular helper cells. Image created with BioRender.

In 1998 (Figure 2), Wada et al. investigated lymphocyte infiltration in hepatocellular carcinoma and observed that lymphatic infiltration was associated with better prognosis. Specifically, the group with marked inflammatory cell infiltration showed a significantly lower recurrence rate (9.1% vs. 47.5%) and higher 5‐year survival rates (100% vs. 65.1%) compared to control groups, providing early evidence for the prognostic role of immune cell infiltration, including TLS in cancer [30, 31].

A decade later, in 2008, Dieu‐Nosjean et al. reported the presence of TLS within tumours but not in non‐cancerous lung tissues, and demonstrated that these tumour‐induced bronchus‐associated lymphoid tissue displayed features that resemble an ongoing immune response [32]. Their innovative approach used TLS characterisation of mature dendritic cells (DCs), which are exclusively ensconced in TLS, as a specific marker, shedding light on the complexity of quantitatively assessing these structures. Importantly, their findings that the density of mature DCs in TLS was highly associated with favourable clinical outcomes support the concept that it is not TLS presence, but their specific cellular and functional features, that serve as the prognostic biomarker.

High densities of mature DCs within TLS were associated with a robust infiltration of effector‐memory T‐cells and improved survival outcomes, reinforcing the importance of TLS in fostering a favourable immune environment [33]. Subsequent research emphasised the role of TLS in shaping the tumour immune landscape, such as fomenting the integration of B‐cell and T‐cell responses, as reflected by prognostically favourable CD8(+) TIL responses in ovarian cancer, only in the presence of plasma cells [34]. TLS support B‐cell maturation into plasma cells with clonal selection and amplification, which then migrate to the tumour bed to exert anti‐tumour activity [17].

However, most studies on the prognostic value of TLSs do not provide a strong characterisation of their features. In NSCLC, the prevalence of TLSs decreases from stage II to stage III, yet the presence of TLS remains independently associated with a favourable prognosis, reinforcing its role as an independent predictor of prognosis [35, 36]. In desmoplastic melanoma, intratumoral TLSs have been associated with increased T‐ and B‐cell densities, as well as high proliferation of CD20^+^ and CD8^+^ cells [37]. Although desmoplastic melanoma had fewer TLS per tumour than non‐desmoplastic melanoma, its immune‐rich composition suggested functional relevance. Additionally, researchers identified 39 TLS‐related genes (TLS‐RGs) to develop a risk model for prognosis in cutaneous melanomas and to stratify patients into low and high‐risk groups independent of disease stage [38]. The developed nomogram demonstrated predictive accuracy (AUC = 0.793); however, reproducibility across independent cohorts is needed to support the clinical utility of TLS‐based prognostic modelling.

Despite these encouraging findings, conflicting evidence exists in other cancers. TLSs have been found to support tumour growth in liver cancer when driven by chronic NF‐κB activation, providing a pro‐tumour environment that promotes tumour progression [39]. CXCL13, a surrogate marker of TLS, has been associated with poor prognosis in renal carcinoma and breast cancer [24, 40, 41, 42]. Moreover, intratumoral TLSs are observed in breast cancers with high immune cell infiltration and are associated with higher tumour grade and inflammation, suggesting their presence may reflect tumour‐promoting inflammatory processes in aggressive breast cancers [43].

Despite these contradictory results, the potential prognostic value of TLS across various cancer types appears strongest when TLS are defined not solely by their presence, but by specific cellular or functional characteristics. This underscores the need for further research to refine their clinical application.

## 4 TLS As a Predictive Biomarker of Response to Immunotherapy

Several studies have shown that the presence of mature intratumoral TLS before immunotherapy treatment is strongly linked to positive outcomes in sarcoma, melanoma, and lung cancer (Figure 2). High immune cell infiltration, including B and T‐cells, has been identified as a strong predictor of improved survival after treatment with immune checkpoint inhibitors (ICI) [44]. However, in tumours with high immune cell infiltration, the effectiveness of anti‐PD‐1 immunotherapy was reduced unless mature TLS with organised immune zones were present, highlighting the importance of TLS organisation for immunotherapy success.

In the study by Cabrita et al. melanoma patients with a TLS gene signature showed improved survival and a better response to ICIs [7]. The presence of TLS not only correlated with increased survival but also played a key role in shaping the tumour microenvironment and modulating immune responses. CXCL13, a TLS marker, was identified as an immunotherapy prognostic marker in a gastric cancer study by Xu et al. aligning with findings in advanced bladder cancer patients [45, 46].

Additionally, responders to ICI exhibit significantly higher densities of CD20^+^ B‐cells and TLS, as well as a greater ratio of TLS to tumour area, especially in early treatment samples compared to non‐responders [47, 48]. This pattern suggests an effective early immune response in those who benefit from the therapy. Moreover, some studies suggest that both the TLS composition and location within tumours are important to orchestrate a functional anti‐tumour response [48, 49].

Mature and intratumorally located TLSs, characterised by the presence of CD21^+^ follicular dendritic cells and CD23^+^ germinal centre B‐cells, play a crucial role in facilitating effective immune responses against cancer [14, 50]. The intratumoral position of these TLS structures allows for direct interaction with tumour cells and greater access to tumour antigens, which can enhance the recruitment and activation of antigen‐presenting cells and effector T‐cells [50]. This proximity potentially optimises the immune‐stimulating environment necessary for better outcomes and may contribute to improved patient prognosis. However, the evidence supporting the greater prognostic significance of intratumoral versus peritumoral TLS remains limited and not widely established, indicating a need for more research to clarify how the location and organisation of TLS impact their functionality and efficacy in the context of cancer immunotherapy.

Vanhersecke et al. investigated the predictive value of mature TLSs, defined by the presence of CD23^+^ follicular dendritic cells within the TLSs, in solid tumours treated with ICI, regardless of PD‐L1 expression [14]. They analysed various tumour samples from 328 patients treated with anti‐PD1 antibodies, among whom 127 were NSCLC patients. The presence of mature TLSs was associated with longer progression‐free survival and overall survival, demonstrating the importance of TLS characterisation for predictive use.

Interestingly, a study by the same group [51] found that the presence of mature TLS did not predict treatment response or progression‐free survival in NSCLC patients undergoing platinum‐based chemotherapy, underscoring the specific relationship between TLS and immune control. Similar findings regarding the lack of association between mature TLS presence and chemotherapy outcomes in NSCLC patients were also noted in another study [52]. The debate over chemotherapy's impact on TLS induction persists, with conflicting evidence regarding its ability to either stimulate or impede TLS formation [51, 53].

Nevertheless, the Neostar phase 2 trial showed that adding the CTLA‐4 inhibitor ipilimumab to neoadjuvant nivolumab and chemotherapy in resectable NSCLC patients increases TLS presence and B‐cell abundance, suggesting a significant role of TLS in enhancing anti‐tumour responses with CTLA‐4 blockade [54]. Similarly, a recent study in hepatocellular carcinoma patients treated with neoadjuvant ICI highlighted that the presence of intratumoral TLS in the resected residual tumour was associated with improved prognosis [55]. Interestingly, the authors described a distinct noncanonical involuted TLS in areas of tumour regression linked to the memory phase of the intratumoral adaptive immune response.

Leveraging the potential of TLS as a biomarker to tailor treatment with immunotherapy, the PEMBROSARC trial (NCT02406781) selected patients with advanced soft‐tissue sarcomas containing TLS for treatment with pembrolizumab combined with low‐dose cyclophosphamide [56]. This selection was based on a previous study by the same group [44], which identified a B‐cell related gene signature that characterised an immune high subgroup of the patients with elevated signature levels, associated with intratumoral TLS. Their outstanding results showed a 6‐month non‐progression rate of 40% and an objective response rate of 30%, compared to 4.9% and 2.4%, respectively, in the all‐comers cohorts [56], cementing TLS presence as a promising biomarker for selecting patients who may benefit from this treatment.

## 5 TLS in Cancer Treatments

Initially studied for their role in immune cell generation, TLS have recently gained attention for their therapeutic relevance in cancer, prompting efforts to induce or manipulate them within the TME [22]. More recently, several studies have delved into different aspects of TLS formation and manipulation, shedding light on various strategies for their therapeutic indication (Table 1) [8, 59, 62, 63, 64].

**TABLE 1:** Summary of TLS induction studies.

Type of study	TLS induction approach	Result	Cancer type	References
Preclinical	LTBR targeting	Enhanced anti‐tumour immune surveillance	Solid tumours	Lukashev et al. [57]
Preclinical	LTBR pathways	Improve immune response	Solid tumours	Peske et al. [58]
Preclinical	LIGHT‐VTP	Normalise tumour vasculature	Pancreatic cancer	Johansson‐Percival et al. [9]
Preclinical	STING Agonist	Normalise tumour vasculature	Melanoma	Chelvanambi et al. [59]
Preclinical	Doxorubicin treatment	Promote HEV and TLS formation	Solid tumours	Zhao et al. [60]
Clinical	HPV vaccination	TLS with effector T‐cells	Cervical cancer	Maldonado et al. [61]
Clinical	PEF	Immunogenic cell death	NSCLC	Iding et al. [8]

Advancements in preclinical research have significantly enhanced our understanding of TLS induction in solid tumours [65]. Various strategies have been explored, such as a mechanistic approach of leveraging signalling pathways like lymphotoxin beta receptor (LTBR), which is critical for the development and maintenance of different lymphoid microenvironments [57, 58]. LTBR activation promotes the differentiation of HEVs, which is essential for lymphocyte trafficking. Furthermore, LTBR signalling has been implicated in facilitating TLS formation in chronically inflamed settings. Therefore, artificially inducing TLS through LTBR activation can aid in enhancing anti‐tumour immune surveillance.

Another innovative approach involves using LIGHT‐VTP, a fusion compound targeting angiogenic tumour vessels, that has shown promise by normalising tumour vasculature and inducing TLS with distinct immune cell zones in neuroendocrine pancreatic models [9]. Similarly, the STING agonist, ADU S‐100 has been found to normalise tumour vasculature, facilitate immune infiltration, and induce TLS in melanoma models, highlighting its potential to reshape the tumour microenvironment and enhance immune responses [59].

Intramuscular vaccination approaches targeting HPV16 E6/E7 antigens are also shown to induce intratumoral TLS in high‐grade cervical intraepithelial neoplasia, characterised by lymphoid aggregates with germinal centres [61]. This demonstrates vaccination can help reshape the tissue microenvironment and induce TLS formation through antigen‐specific immune activation.

Transitioning to clinical research, a notable study by Iding et al. investigated TLS formation in NSCLC tumours following physical electroporation of tumours (PEF) using the Aliya system [8]. This approach is performed either percutaneously or endoscopically at the time of biopsy before surgical resection. The findings highlighted PEF‐induced immunogenic cell death as a trigger for TLS formation [8], aligning with broader research on immunogenic death, stimulating immune responses and TLS development [60]. Understanding the intricate interplay between cell death pathways, immune activation, and TLS formation is crucial for refining therapeutic approaches.

Overall, these advancements underscore the growing promise of TLS induction in cancer immunotherapy, highlighting both the progress made in preclinical research and the emerging clinical applications. Continued research is essential to refine and translate these strategies into effective treatments that leverage immune responses within the tumour microenvironment.

## 6 Conclusions and Future Perspectives

Overall, recent advances highlight the growing promise of TLS induction in cancer immunotherapy, with notable progress in preclinical studies and emerging clinical applications. These findings support the potential of TLS as modulators of anti‐tumour immunity and as biomarkers of therapeutic response.

Despite these promises, clinical translation remains limited by several methodological and biological challenges. TLS are inherently heterogeneous, vary across disease stages, and can exert both pro‐ and anti‐tumour effects, making their role difficult to standardise in clinical protocols. Their dual capacity to either potentiate or inhibit tumour progression introduces complexity in leveraging TLS for therapeutic purposes.

Current detection methods, including immunohistochemistry, multiplex immunohistochemistry, multiplex immunofluorescence, and transcriptional profiling, are informative but largely dependent on surgical specimens [11, 66]. Their reliability in core biopsies and fine needle aspirates remains uncertain, and standardisation issues persist, limiting broader clinical application [55].

To realise their full potential in precision oncology, further research is needed on optimising TLS identification, understanding their immunological dynamics across disease contexts and developing safe, non‐invasive monitoring strategies suited for clinical use. This will aid in overcoming current barriers and enhance the clinical utility of TLS in cancer treatment.

In conclusion, continued exploration into the biological mechanisms underpinning TLS formation, maturity, and immune function is critical. Moreover, refining detection methods, coupled with efforts to standardise TLS metrics, will be vital to harnessing their prognostic and therapeutic potential in oncology.

## Author Contributions

**Lidia B. Medhin:** conceptualization (lead), data curation (lead), investigation (lead), writing – original draft (lead), writing – review and editing (lead). **Rodas Amanuel:** data curation (supporting), writing – original draft (supporting). **Lydia Warburton:** writing – review and editing (equal). **Leslie Calapre:** writing – review and editing (equal). **Benhur Amanuel:** conceptualization (equal), supervision (equal), writing – review and editing (equal). **Elin S. Gray:** conceptualization (equal), data curation (equal), supervision (lead), writing – original draft (equal), writing – review and editing (equal).

## Funding

This work was supported by Raine Medical Research Foundation.

## Conflicts of Interest

The authors declare no conflicts of interest.

## Data Availability

Data sharing not applicable to this article as no datasets were generated or analysed during the current study.
